# Silencing of X-Linked MicroRNAs by Meiotic Sex Chromosome Inactivation

**DOI:** 10.1371/journal.pgen.1005461

**Published:** 2015-10-28

**Authors:** Hélène Royo, Hervé Seitz, Elias ElInati, Antoine H. F. M. Peters, Michael B. Stadler, James M. A. Turner

**Affiliations:** 1 Friedrich Miescher Institute for Biomedical Research (FMI), Basel, Switzerland; 2 Swiss Institute of Bioinformatics, Basel, Switzerland; 3 The Francis Crick Institute, Mill Hill Laboratory, London, United Kingdom; 4 Institute of Human Genetics, UPR 1142, CNRS, Montpellier, France; Cornell University, UNITED STATES

## Abstract

During the pachytene stage of meiosis in male mammals, the X and Y chromosomes are transcriptionally silenced by Meiotic Sex Chromosome Inactivation (MSCI). MSCI is conserved in therian mammals and is essential for normal male fertility. Transcriptomics approaches have demonstrated that in mice, most or all protein-coding genes on the X chromosome are subject to MSCI. However, it is unclear whether X-linked non-coding RNAs behave in a similar manner. The X chromosome is enriched in microRNA (miRNA) genes, with many exhibiting testis-biased expression. Importantly, high expression levels of X-linked miRNAs (X-miRNAs) have been reported in pachytene spermatocytes, indicating that these genes may escape MSCI, and perhaps play a role in the XY-silencing process. Here we use RNA FISH to examine X-miRNA expression in the male germ line. We find that, like protein-coding X-genes, X-miRNAs are expressed prior to prophase I and are thereafter silenced during pachynema. X-miRNA silencing does not occur in mouse models with defective MSCI. Furthermore, X-miRNAs are expressed at pachynema when present as autosomally integrated transgenes. Thus, we conclude that silencing of X-miRNAs during pachynema in wild type males is MSCI-dependent. Importantly, misexpression of X-miRNAs during pachynema causes spermatogenic defects. We propose that MSCI represents a chromosomal mechanism by which X-miRNAs, and other potential X-encoded repressors, can be silenced, thereby regulating genes with critical late spermatogenic functions.

## Author Summary

During male germ cell formation, the X and the Y chromosomes are inactivated. This process is conserved and it is essential for germ cell generation. It is believed that X/Y silencing affects all protein-coding genes, but the status of miRNAs and other non-coding genes needs further investigation. MicroRNAs from the X-chromosome (X-miRNAs) have been reported as potential silencing escapers, and they have been proposed to play a role in the inactivation mechanism itself. By looking at the individual cell level, we show unambiguously that X-miRNAs are subject to X/Y silencing, a finding that contradicts the current literature. Moreover, we generated mouse mutants in which we forced expression of X-miRNAs during X/Y silencing, and this lead to germ cell death. We propose that X/Y silencing can influence transcription of essential germ cell genes by regulating X-repressors.

## Introduction

Meiotic sex chromosome inactivation (MSCI) describes the transcriptional silencing of the unsynapsed X and Y chromosomes at the onset of pachynema in mammalian male germ cells [1–5]. Inactivation of the sex chromosome results in the formation of a heterochromatic domain called the sex body [6]. MSCI is one example of a general mechanism, meiotic silencing, which inactivates any chromosome that is unsynapsed during male or female meiosis [7,8]. MSCI imposes a repressive chromatin signature on the X and Y chromosomes that is retained later, during spermiogenesis [9–12]. MSCI and its maintenance are regulated by a broad array of DNA double-strand break (DSB) repair proteins and chromatin modifications [2,5,13]. Male mice with chromosome abnormalities, e.g. XYY, or targeted mutations in meiotic synapsis or recombination genes, e.g. *Spo11-/-* and *Brca1-/-* frequently exhibit defective MSCI, and this results in misexpression of toxic sex-linked genes and midpachytene arrest [14–18].

Microarray [9,19], RNA-sequencing [20] and RNA FISH [16] studies have concluded that in mice MSCI is robust, and no example of an X-linked protein-coding gene that is actively transcribed during pachynema has yet been identified. This situation contrasts with that later in spermatogenesis, when expression of some X-linked genes from the repressed X chromosome is facilitated by various mechanisms including gene amplification [16] and establishment of active chromatin marks by the ubiquitin ligase RNF8 [21]. However, the activity of X-derived non-coding RNAs, and especially small RNAs, during pachynema is less well understood.

Interestingly, the X mouse chromosome is enriched in miRNA-encoding genes, and many of these are expressed in a testis-biased manner [22–24]. Song et al. conducted an extensive study of X- miRNA expression patterns, in which miRNA levels were evaluated by RT-qPCR in purified spermatogenic cell populations. 86% of X-linked miRNA transcripts were detected at high levels in pachytene spermatocytes, and it was been suggested that these genes escape MSCI [25]. High pachytene levels of X-miRNA transcripts have been confirmed by RT-qPCR [22,25], RNA-sequencing [23,26,27] and *in situ* hybridisation [25,27] approaches. Non-coding RNAs have a prominent role in gene silencing, e.g. in X chromosome inactivation [28], and repression of transposable elements and centromeric repeats [29], and it is therefore possible that X-linked miRNAs contribute to the process of MSCI itself. However, definitive proof that X-miRNA genes escape MSCI requires that nascent precursors of miRNAs, so-called pri-miRNAs, are generated during pachynema, and ideally, that these can be visualised as nascent transcripts originating from the otherwise inactive X chromosome, e.g. by techniques such as RNA FISH. We therefore sought to reappraise X-miRNA expression in the male germ line focusing on nascent transcripts.

## Results

### X-miRNAs are silenced during pachynema

**Fig 1:**
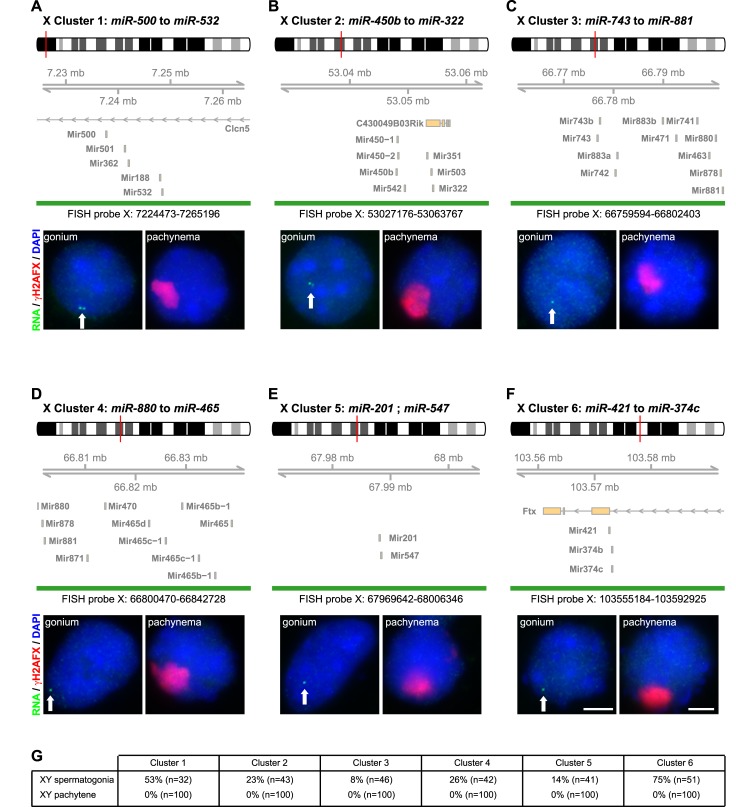
X-linked miRNA precursors are not expressed at pachynema. A-F) In each panel, the fosmid probes used for the detection of miRNA precursors by RNA FISH for 6 miRNA clusters are shown (green bars). An ideogram of the X chromosome is displayed (top, fosmid position indicated as a thin vertical red line), together with the genomic coordinates of the fosmid, and the UCSC genes reported in the region (middle). Bottom: Representative RNA FISH image of a pachytene spermatocyte (right; no expression); a spermatogonia is shown as a control (left, the arrows point to a positive signal). Red: γH2AFX, green: RNA FISH signal, blue: DAPI. Cells were recognised based on γH2AFX staining and DAPI appearance. Scale bars: 5μm; scale bars displayed in F apply to A-F. G) Quantitative analysis of RNA FISH data. The fraction of miRNA-expressing spermatogonia varies from one cluster to the other, probably reflecting different expression levels and cell-to-cell heterogeneity in gene expression programs or developmental stages.

In order to establish whether X-miRNAs are subject to MSCI, we examined their expression during mouse spermatogenesis using RNA FISH (Fig 1A–1G). We focused on spermatogonia, the early diploid germ cell progenitors in which the X chromosome is active, and pachytene spermatocytes, in which MSCI has taken place. There are currently 167 annotated miRNA genes on the X chromosome (source: miRBase version 21), the majority of which fall into clusters. We focused on six clusters, located at different sites on the X chromosome, and expressed in the testis (S1 Fig) [23]. Together these comprise 78 X-miRNAs, and 83% of them have been reported to escape MSCI [25]. We used a combination of antibody staining for the MSCI marker phosphorylated histone H2AFX (γH2AFX) [30], as well as DAPI nuclear staining, in order to accurately substage germ cells.

X-miRNA clusters 1 and 6 reside within introns of the genes *Clcn5* and *Ftx*, respectively, and are transcribed from the same strand as the host genes (Fig 1A and 1F). At miRNA cluster 1 and cluster 6 loci, no putative promoter other than the host gene promoters can be detected upstream the miRNA genes, as assessed by H3K4me3 signal (S2 Fig), and expression of the miRNAs was shown to be dependent on the transcription of *Clcn5* and *Ftx* parental RNAs [31,32]. *Clcn5* and *Ftx* primary transcripts therefore represent the X-miRNA precursor transcripts. We used fluorescently-labelled, denatured fosmid DNA probes spanning the intronic *Clcn5* and *Ftx* X-miRNA containing regions in order to detect X-miRNA precursor transcripts (X-pri-miRNA). Cluster 1 and 6 pri-miRNA FISH signals were observed in spermatogonia (53% and 75% expressing, n = 32 and 51 cells, respectively). However, no pri-miRNA expression could be detected in pachytene spermatocytes (0% expressing, n = 100 cells; Fig 1A, 1F and 1G).

Next, we used fosmid probes to examine expression of the remaining X-miRNA clusters 2, 3, 4 and 5. X-miRNAs located within these clusters do not lie within host genes. For all four clusters, we observed pri-miRNA FISH signals in spermatogonia (23%, 8%, 26% and 14% expressing, n = 43, 46, 42 and 41 cells, respectively; Fig 1B–1E and 1G). In contrast, RNA FISH signals were not observed in pachytene spermatocytes for any of the four clusters (0% expressing for each cluster, n = 100 cells each in each case; Fig 1B–1E and 1G).

The fosmids that we used for our RNA FISH experiments have an average size of 39kb. These probes will detect X-miRNA transcription, but could potentially also detect unannotated transcripts residing in the same locus. To exclude this possibility, we carried out two experiments. For cluster 5, we used recombineering to excise a 7kb segment containing the X-miRNA genes from the fosmid probe. When the resulting, modified fosmid was used for RNA FISH, no signals were observed in spermatogonia (0% displaying signals, n = 41 cells; S3 Fig). Secondly, we designed an RNA FISH protocol to assess transcription of specific X-pri-miRNAs. In this approach, we used ~40 nucleotide-long probes matching sequences present in the pri-miRNA, but not the pre-miRNA or the mature miRNA, at the base of the miRNA-containing stem-loop sequence (S4 Fig). We targeted the X-miRNA miR-465, present in six copies in cluster 4 (Fig 1D). Pri-miRNA signals were observed in spermatogonia but not in pachytene cells (0% expressing, n = 53 cells; S4 Fig). Thus, in conclusion, we observed transcription of all six X-miRNA clusters (total 78 X-miRNAs) in spermatogonia. However, we could not detect expression for any of these X-miRNAs during pachynema.

### X-miRNAs are subject to MSCI

**Fig 2:**
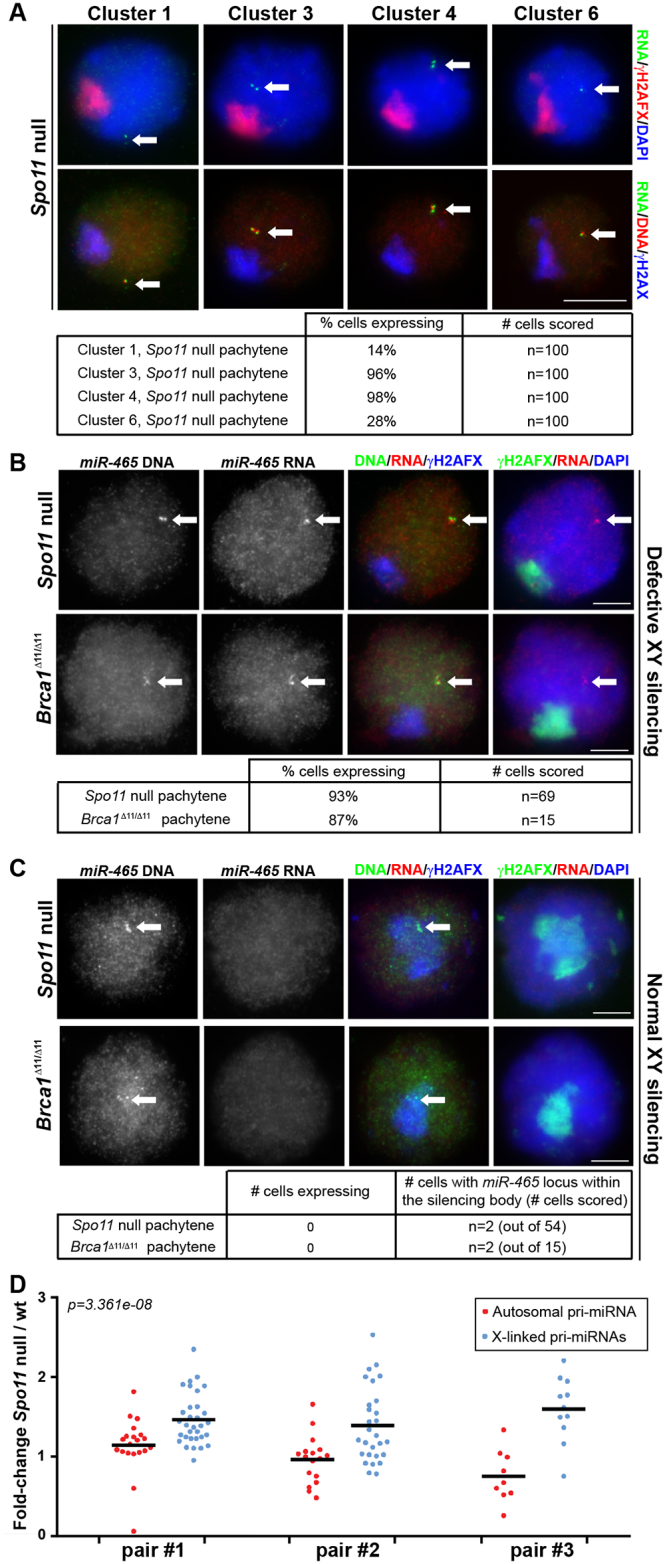
Pachytene silencing of X-linked miRNAs is due to MSCI. A) Expression of miRNA precursors was tested for miRNA clusters 1, 3, 4 and 6 by combined DNA/RNA FISH using fosmids in *Spo11* null males, which exhibit defective MSCI. Precursors for all four miRNA clusters were detected in *Spo11* null pachytene spermatocytes. B) Pri-miRNA FISH images of pachytene cells expressing *miR-465* in *Spo11* null and *Brca1* ^*Δ11/Δ11*^ mutants (defective in XY silencing). Pri-miR-465-specific oligonucleotide probes and BAC probes were used for RNA and DNA FISH respectively. C) In some rare pachytene cell, the *miR-465* locus is found underneath the silencing body that forms in *Spo11* null and *Brca1* ^*Δ11/Δ11*^ spermatocytes. This affects less than 5% and 15% of the *Spo11* null and *Brca1* ^*Δ11/Δ11*^ spermatocytes, respectively. *MiR-465* is silenced in those instances. A-C) Arrows point to positive FISH signals. Tables: quantitative analysis of RNA FISH data. Scale bars: 5 μm; scale bar in A applies to all images in A. D) Expression of individual X-linked versus autosomal pri-miRNAs was tested by Taqman RT-qPCR. The plot shows expression ratio between *Spo11* null over wild type testis of 15.5d*pp* mice. Because most spermatocytes are in pachynema at that stage, genes normally subject to pachytene silencing are expected to be overexpressed in *Spo11* null 15.5d*pp* testis compared to wild type. Each dot represents one X-linked (blue) or autosomal (red) pri-miRNA. The bars show average autosomal or X-linked pri-miRNA expression. Three pairs of *Spo11* null / wild type siblings were tested (pair #1, #2 and #3). X-linked pri-miRNAs are more affected than autosomal pri-miRNAs by the *Spo11* mutation (Wilcoxon test p-value on the pooled three pairs of littermates is displayed).

The absence of cluster 1 to 6 X-pri-miRNA FISH signals in pachytene spermatocytes suggests that these genes are subject to MSCI. To test this possibility, we repeated our RNA FISH analysis on a mouse model in which MSCI is defective. In *Spo11* null male mice, a domain of γH2AFX is formed at pachynema, but this rarely encompasses the X and Y chromosomes, and it is therefore termed the “pseudo sex body” [15,33]. The failure to execute H2AFX phosphorylation on the XY bivalent causes misexpression of sex-linked genes during pachynema in this mutant [16]. We performed pri-miRNA FISH for four representative X-miRNA clusters: 1, 3, 4 and 6 (Fig 2). Pachytene spermatocytes were identified in *Spo11* null males by the presence of the γH2AFX-labelled pseudo sex body.

Notably, in *Spo11* null pachytene spermatocytes we observed pri-miRNA FISH signals for all four gene clusters studied. Expression was observed in 14%, 96%, 98% and 28% of pachytene cells for clusters 1, 3, 4 and 6, respectively (n = 100 cells in each case; Fig 2A). We subsequently repeated the analysis of cluster 4 X-miRNAs using our oligonucleotide RNA FISH approach that specifically detects pri-miRNAs for the six copies of miR-465. We observed FISH pri-miRNA signals in 93% of *Spo11* null pachytene cells (n = 69 cells; Fig 2B). In addition, we performed miR-465-specific pri-miRNA FISH in a second MSCI mutant, the *Brca1* ^Δ11/Δ11^ model [17,18]. We detected misexpression of this miRNA in 87% of pachytene spermatocytes. (n = 15 cells; Fig 2B). We conclude that defective MSCI leads to X-miRNA misexpression during pachynema.

Although MSCI is defective in most *Spo11-/-* and *Brca1* ^Δ11/Δ11^ pachytene cells, in both models domains of γH2AFX are occasionally seen covering sub-regions of the X chromosome [16,17]. We predicted that in these rare spermatocytes, X-miRNAs encompassed within γH2AFX regions should be normally silenced. This proved to be the case: in the few *Spo11* null and *Brca1* pachytene cells in which the miR-465 locus, identified using DNA FISH, lay within a γH2AFX domain (*Spo11* null: n = 2 out of 54 cells; *Brca1* ^Δ11/Δ11^: n = 2 out of 15 cells), no pri-miR-465 expression could be observed (Fig 2C). Thus, the expression status of these X-miRNAs is tightly linked to the presence of the meiotic silencing marker γH2AFX. We conclude that in wild type pachytene spermatocytes, the X-linked miRNAs studied herein are silenced during pachynema as a result of MSCI.

To corroborate our X-miRNA FISH data, we next compared expression levels of individual pri-miRNAs in wild type and *Spo11* null sibling testes by RT-qPCR at 15.5days *post-partum* (d*pp*; Fig 2D). At this age, most spermatocytes are in pachynema, and genes subject to MSCI are expected to be overexpressed in *Spo11* null relative to wild type males. We examined transcript levels for a number of X-linked and autosomal pri-miRNAs, and expressed these as a *Spo11* null / wild type ratio. Experiments were performed in triplicate, each time using a different *Spo11* null and wild type sibling. In each case, *Spo11* null / wild type ratios for autosomal miRNAs averaged ca. 1, indicating no difference in pachytene expression levels between the two genotypes (Fig 2D). Conversely, the ratio for X-linked pri-miRNAs significantly exceeded one (p = 3.361e-08; Fig 2D), thereby confirming that X-linked miRNAs are upregulated in the absence of MSCI.

**Fig 3:**
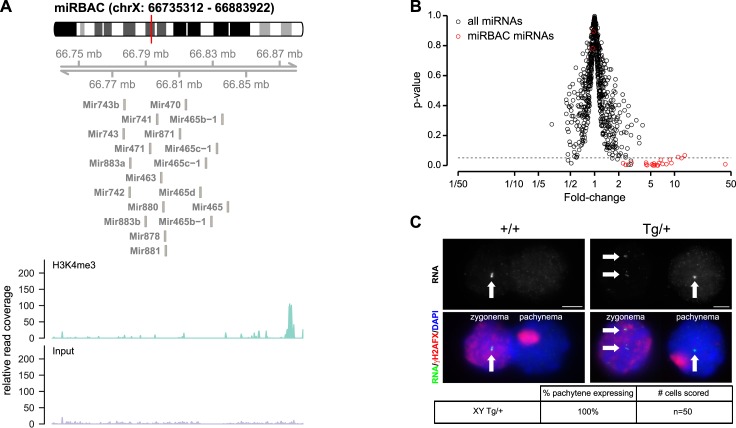
Pachytene expression of X-linked miRNAs from an autosomal transgene. A) Top: Genomic location of the BAC transgene used for the generation of X-miRBAC transgenic mice. The transgene contains no other genes than the miRNA genes from clusters 3 and 4 (source: UCSC). Bottom: H3K4me3 occupancy in spermatogonia at the miRBAC genomic region. ChIP seq profiles are shown for H3K4me3 ChIP and a control sample. The H3K4me3 profile shows a region of strong enrichment upstream of the miRNA cluster (all miRNAs displayed are on the—strand (source: UCSC)), which may represent the promoter region for the miRNA locus. B) Volcano plot showing miRNA expression ratio between transgenic and wild type testis (Fold-change, x-axis), and their significance (p-value, y-axis), as measured by Affymetrix microarrays. The dashed line represents a p-value of 0.05. MiRNAs significantly overexpressed in the transgenic testis are those contained in the X-miRBAC transgene (shown in red). C) Fosmid RNA FISH images of cluster 3/4 miRNA expression in wild type (left) and miRBAC line 1 transgenic (right) males. One zygonema and one pachynema cell are shown. In both genotypes, cluster 3/4 miRNAs are expressed from the X chromosome at zygonema and are silenced in pachynema. However, in the transgenic male, the transgenic cluster 3/4 miRNAs continue to be expressed during pachynema. Table: quantitative analysis of RNA FISH.

Finally, we used transgenesis to further investigate whether silencing of X-miRNAs in pachynema is due to MSCI. Previous experiments have demonstrated that X-genes present as transgenes on autosomes continue to be expressed during pachynema [34]. This is because unlike the X chromosome, autosomes are synapsed during pachynema and therefore escape the effects of meiotic silencing. To establish whether pachytene silencing of X-linked miRNA genes was due to their location on the X chromosome, we generated a single copy transgenic line in which X-linked miRNA gene clusters 3 and 4 were located together on an autosome by random BAC integration (X-miRBAC line 1; Fig 3A). We chose a BAC that includes a region of local H3K4me3 enrichment upstream of the miRNA gene cluster, indicative of a putative promoter (Fig 3A). Using pri-miRNA microarrays, we confirmed that cluster 3 / 4 X-miRNAs were overexpressed in X-miRBAC line 1 testes relative to non-transgenic siblings (Fig 3B). We then performed pri-miRNA FISH in pachytene spermatocytes from X-miRBAC line 1 transgenics using BAC probes covering clusters 3 and 4. We observed expression of cluster 3/4 miRNAs from both the X chromosome and the autosomal transgene prior to pachynema (Fig 3C). However, during pachynema, while the X-located 3/4 miRNAs were silenced, those located on the transgene continued to express (100%, n = 50). We observed the same results using our miR-465-specific pri-miRNA FISH protocol on X-miRBAC line 1 (S5 Fig) (100%, n = 9). Thus, silencing of X-integrated miRNAs during pachynema is due to MSCI.

### Pachytene X-miRNA expression causes spermatogenic defects

**Fig 4:**
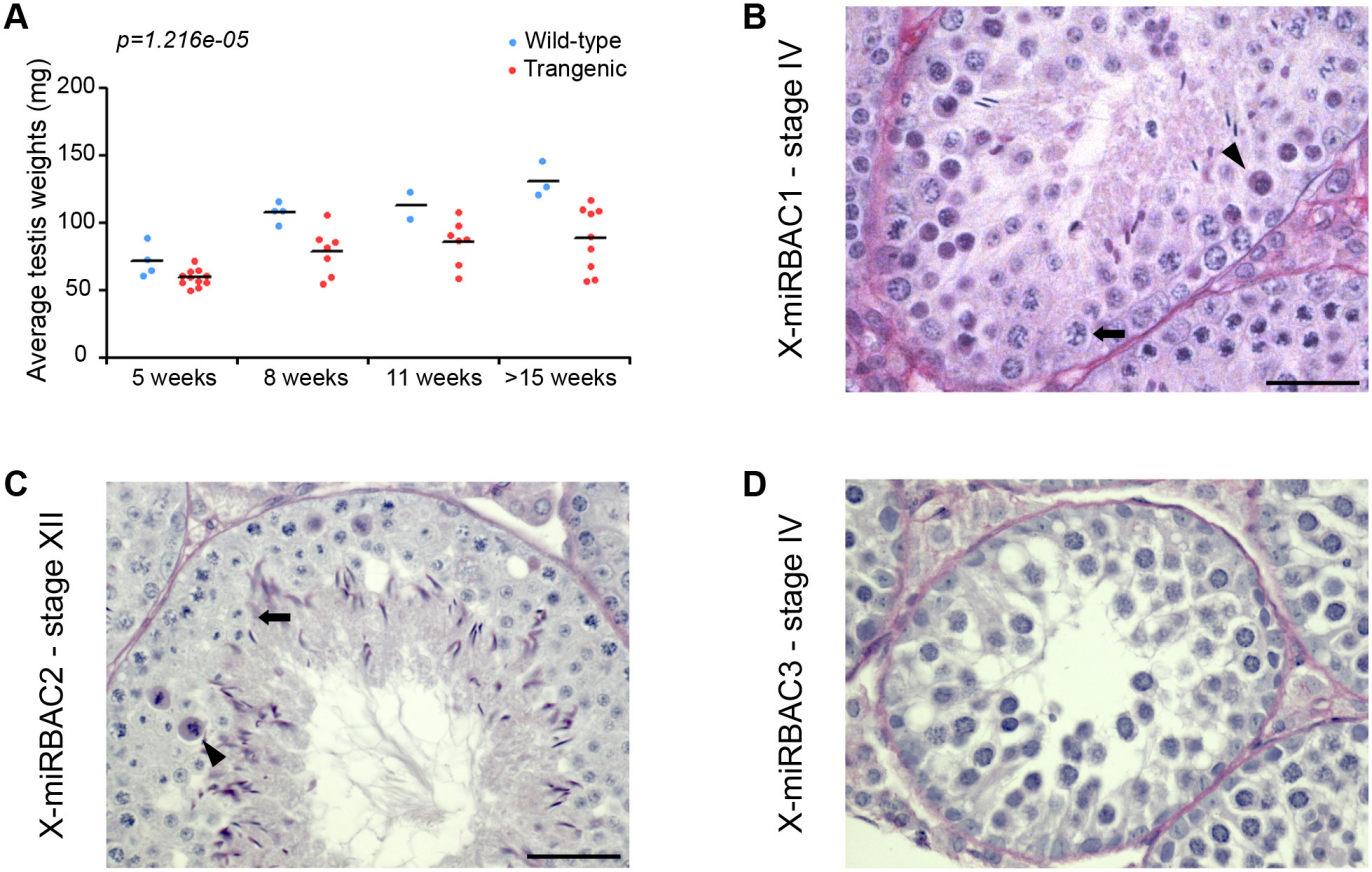
Pachytene silencing of X-linked miRNAs is necessary for completion of meiosis. A) Top: testis weights for wild type (blue) versus X-miRBAC1 transgenic (red) siblings. Each data point represents the average of the left and right testis weight for one individual. Testis from transgenic males are significantly lower than testis from wild type males (two-way ANOVA p-value for the effect of genotype is displayed). B) MiRBAC line 1 testis section stained with PAS showing stage IV cell death. C) MiRBAC line 2 testis section stained with PAS showing stage XII cell death. D) MiRBAC line 3 testis section stained with PAS showing stage IV cell death. The arrowheads and arrows point to dying and healthy spermatocytes respectively. Scale bar: 20μm.

Defects in MSCI cause pachytene arrest, due to misexpression of toxic sex-linked genes, e.g. the Y chromosome genes *Zfy1* and *Zfy2* [14]. Our analyses indicated that X-miRNAs are subject to MSCI. We therefore wondered whether misexpression of these genes during pachynema would give rise to spermatogenic defects. Interestingly, in our X-miRBAC line 1 males, which carry the autosomally-integrated single copy X-miRNA 3 and 4 cluster transgene, we observed reduced testis weights relative to non-transgenic brothers from as early as five weeks post-partum (Fig 4A). Importantly, histological and TUNEL analysis of X-miRBAC line 1 testis sections revealed spermatogenic defects, principally germ cell apoptosis at stage IV, corresponding to midpachynema, and stage XII, corresponding to the meiotic divisions (Fig 4B, S7 Fig).

In order to exclude the possibility that the spermatogenic defects observed in X-mirBAC line 1 males resulted from a transgene integration effects, we subsequently generated two more cluster 3/4 X-miRNA autosomal transgenic lines, with three (X-miRBAC line 2) and eleven (X-miRBAC line 3) transgene copies (S6 Fig). X-miRBAC line 2 showed predominant apoptosis at stage XII (Fig 4C, S7 Fig), while X-miRBAC line 3 exhibited marked apoptosis at mid and late pachynema (Fig 4D, S7 Fig). We conclude that inappropriate expression of X-miRNAs from the X-linked clusters 3 and 4 at pachynema induces spermatogenic defects.

## Discussion

MSCI is a robust silencing process, affecting most or all protein-coding genes on the mouse X chromosome. However, it is unclear whether silencing also affects X-linked miRNAs. Here, using RNA FISH and other transcriptional assays, we find that X-linked miRNA genes are expressed before prophase I but are silent during pachynema. We therefore conclude that X-miRNAs are subject to MSCI.

It is important to highlight that we did not study all miRNAs on the X chromosome. It is therefore formally possible that X-miRNAs omitted in our analyses behave differently with respect to MSCI. We find this unlikely, because we chose miRNAs from multiple, distinct clusters on the X chromosome, and we included many X-miRNAs that were previously reported to escape silencing [25]. *A priori*, one could also argue that our inability to detect X-miRNA FISH signals during pachynema is because our RNA FISH experiments lack the sensitivity required to detect gene expression during this stage of prophase I, rather than because these genes are subject to MSCI. We doubt that this is the case, because we were able to detect expression of these species during pachynema both in MSCI mutants, and in mice carrying autosomally-located X-miRNA transgenes. Finally, our conclusion that X-miRNAs are subject to MSCI was corroborated by RT-qPCR analysis in MSCI mutants versus controls. Taken together, our data support a model in which X-miRNAs behave like X-linked protein-coding genes with respect to X silencing.

How can our findings accommodate earlier work? Several independent reports have documented high levels of X-miRNA expression in pachytene spermatocytes [22,23,25,26], and there can be little doubt that this contrasts with the generally low level expression detected for protein-coding X-genes. However, in most existing studies X-miRNAs were assayed at the level of mature miRNAs. Notably, with the exception of some rare cases [35,36], miRNAs have an unusually long half-life, on average 5 days, which exceeds that of protein-coding RNAs by ten-fold [37]. The abundant expression of miRNAs during pachynema might therefore be due to their high transcript stability, rather than ongoing generation of nascent miRNA precursors.

Interestingly, our work shows not only that X-miRNAs are subject to MSCI, but also that failure to silence them can result in spermatogenic defects which are manifest in the case of the cluster 3/4 X-miRNAs as arrest predominantly at stages IV and XII. Thus, X-miRNAs join the Y-encoded *Zfy1*/*2* genes as being male “pachytene-lethal” genes. Our findings show that MSCI must be extensive, silencing genes not only on the Y chromosome but also on the X chromosome. Given that miRNAs act as gene repressors, the phenotypes resulting from their ongoing expression in our miRNA transgenic males are presumably due to inappropriate target downregulation as a consequence of miRNA overexpression, or to an inability to appropriately upregulate target genes with meiotic and/or post-meiotic functions. In this model, MSCI could function as a chromosome-based mechanism for regulating expression of repressors. From a broader perspective, the large-scale silencing of genes across the X chromosome by MSCI is likely to influence myriad transcriptomic networks within germ cells. As a consequence, MSCI could regulate multiple facets of the mammalian germ cell development program.

## Materials and Methods

### Ethics statement

All animal procedures were in accordance with the United Kingdom Animal Scientific Procedures Act 1986 and were subject to local ethical review.

### Mice

All mice were maintained on an MF1 background. The miRBAC transgenic lines were produced by microinjection of purified BAC BMQ-333E20 into fertilized eggs from CBA/Ca x C57Bl/10 F1s. *Spo11* null and *Brca1* ^Δ11/Δ11^ mice have been described previously [18,38].

### Genome browser tracks

Browser tracks were generated in R using the Givz package. The UCSC mouse genome assembly mm10 was used as a basis for analysis. Annotation of transcripts was obtained from the UCSC knownGene database. Genomic coordinates of fosmid and BAC probes were obtained from the CHORI BACPAC resource center.

### RNAseq analysis

A small RNA testis library was downloaded from GEO under accession identifier GSE40499 (Meunier et al.). Adapter sequences were removed from the reads (ATCTCGTATGCCGTCTTCTGCTTG), and 15 to 23nt-long reads were selected for analysis. Reads were aligned to the mouse mm10 genome using Bowtie (version 1.6.0) with the parameters -m 50—best—strata -v 2. MiRNA gene coordinates were obtained from miRBAse (version 21). MiRNA duplicates sharing a copy on the X chromosome and a copy on an autosome were removed from the analysis. MiRNA read counts were generated in R using the QuasR package as documented in the reference manual [39]. Read counts are expressed as read counts per million reads mapping to miRNA genes.

### FISH and immunofluorescence

RNA and DNA FISH was carried out with digoxigenin- and biotin-labelled probes respectively, using fosmid and BAC genomic clones (cluster 1: WI1-603H11; cluster 2: WI1-1995I23; cluster 3: WI1-1646F11; cluster 4: WI1-2045C16; cluster 5: WI1-2828J23; cluster 6: WI1-2859G17; miRBAC: BMQ-333E20). The technique was described previously [40]. For miR-465-specific pri-miRNA FISH, a mix of nine amino-allyl-modified oligonucleotides labeled with fluorolink Cy3 were used as probes (S1 Table). We used the anti-γH2AFX antibody (Upstate, 16–193; dilution 1/100) for immunofluorescence post-RNA FISH.

### RT-qPCR

Total RNA was extracted from frozen testis tissues with Trizol (Invitrogen), treated with DNAse I (Invitrogen), and reverse transcribed with random hexamers (Invitrogen) and Superscript II (Invitrogen) according to the manufacturer’s instructions. Quantitative PCR was performed with pre-designed Taqman pri-miRNA and U6 snRNA Taqman assays according to the manufacturer's instructions. Relative expression was calculated with the ΔCt method using U6 as a normaliser. Pri-miRNA expression ratio for individual pri-miRNAs are provided in S2 Table.

### ChIPseq analysis

A library of H3K4me3 ChIP in adult germline precursor cells and corresponding input controls were downloaded from GEO under accession identifier GSE49624 [41]. Reads were aligned to the mouse mm10 genome using Bowtie (version 1.6.0) with the parameters Bowtie -m 1—best –strata.

### Microarray

Affymetrix Mouse miRNA 2.0 microarrays were performed to measure miRNA expression in testis of three wild type and two homozygous transgenic siblings at 37d*pp*. Total RNA was extracted with Trizol (Invitrogen), treated with DNAse I (Invitrogen), and column-purified (Ambion). Microarray hybridizations were performed according to the manufacturer's instructions. Microarray signal intensity was extracted and normalized using Affymetrix' miRNA QC Tool using default parameters. Statistical analyses were performed using R and the limma package. Fold-changes and FDR-adjusted p-values were computed by fitting a linear model for each microRNA. Standard errors were smoothed using empirical Bayes (eBayes function of the limma package).

### Transgene copy number estimation

The transgene copy number of the miRNA BAC transgenic line was estimated by qPCR on genomic DNA, with a technique adapted from the one described in. The data was normalised with *Atr* PCR for ΔCt calculations, and quantification of *Jun* copy number was used as a quality control. Primer sequences are provided in S3 Table.

### Recombineering

Recombination-mediated genetic engineering of fosmid WI1-2828J23 was performed to delete a 7kb fragment encompassing miRNA genes *miR-201* and *miR-547* using standard procedures. The primers used for recombineering are

LN_X8-F (taactagtaagtctaatatattgttgtttaaaacctactgctttgtgctcggcctggtgatgatggcgggatcgttg)
LN_X8-R (ggcccaatgaattatattttctagtacctctcagtatacaaatcaccaactcagaagaactcgtcaagaaggcgata).

## Supporting Information

S1 Fig Expression of X-linked miRNAs in adult testis. Normalized expression of X-linked miRNAs detected by RNA-sequencing in the testis are plotted against their genomic coordinates. Dots correspond to individual miRNAs. Positions of the fosmid probes used in RNA FISH are shown on top. The miRNAs targeted by RNA FISH are coloured by cluster. MiRNAs outside the clusters are in semi-transparent grey (overlapping miRNAs appear as darker data points).
(TIF)

S2 Fig No evidence for miRNA-specific promoters at clusters 1 and 6. H3K4me3 occupancy in spermatogonia at the *Clcn5* (A) and *Ftx* (B) loci. H3K4me3 peaks are detected at the 5’ end of the miRNA host genes *Clcn5* and *Ftx*, and likely represent the promoters driving transcription of these genes. No other putative promoter is detected downstream the miRNAs (miRNAs are encoded on the—strand; dashed line in B shows where the miRNAs map on the ChIP tracks).
(EPS)

S3 Fig Fosmid RNA FISH enables the specific detection of pri-miRNAs. Top: Fosmid probe used for the detection of cluster 5 miRNA precursors by RNA FISH (probe 'Cluster 5'). A probe deleted for the miRNA genes was generated by recombineering (probe 'Cluster 5 Δ'). Bottom: a positive RNA FISH signal is detected with the original fosmid probe in spermatogonia (left panel, arrow). No signal is detected with the deletion probe (right panel). The RNA FISH experiment was performed in parallel with that of fig 1E. Green: RNA FISH signal, blue: DAPI. Scale bar: 5μm.
(TIF)

S4 Fig Pri-miRNA FISH shows that *miR-465* is silent in wild type pachytene cells. Top: Four variants of the *miR-465* gene are repeated in the genome at the XA7 locus (see Fig 1D). Global DNA alignment of the precursors of the miR-465 variants is shown. Areas of high similarity (90% or more) are displayed in blue (source: CloneManager). Five pri-miRNA FISH probes were designed to target conserved sequences at the base of miRNA-containing stem-loop sequence. Middle: All five pri-miRNA probes were mixed for detection of pri-miRNA transcripts by FISH. BAC probes were used for DNA FISH. FISH signals are indicated by arrows. MiR-465 precursors were not detected in pachytene cells. MiR-465 expression pattern recapitulates that of cluster 4 miRNAs (Fig 1D). Note that pri-miRNA FISH signals were weaker than those obtained by fosmid RNA FISH. Scale bars: 5 μm. Bottom: Quantitative analysis of pri-miRNA FISH data for pachytene cells.
(TIF)

S5 Fig Detection of pri-miR-465 by pri-miRNA FISH in miRBAC line 1 transgenic pachytene spermatocytes. Combined DNA / pri-miRNA FISH for miR-465 shows expression of *miR-465* from the autosomal, transgenic locus (arrows) but not from the X locus (arrowhead). Both the transgenic (arrows) and endogenous loci (arrowhead) are detected by DNA FISH. The insets show enhanced images of the endogenous and transgenic loci. Scale bar: 5 μm.
(TIF)

S6 Fig Transgene copy number estimation in the X-miRBAC transgenic lines. The number of copies of the miRNA region spanned by the transgene was estimated by qPCR on genomic DNA of heterozygous transgenic males. As a control, quantification was also made in a female (known number of copies of two). The transgene copy number was first expressed as the ratio between the number of copies in the tested sample (XX control or XY transgenic) and in an XY male. Results are expressed as the average +/- standard deviation of values obtained from three different genomic DNA dilutions. The number of copies of the transgene was then estimated by subtracting the number of copies of the miRNA cluster by the number of endogenous copies (one copy for XY, two for XX). As an additional control, we quantified the number of copies of the autosomal gene *Jun* (known copy number of two).
(TIF)

S7 Fig Phenotypic characterization of X-miRBAC transgenic lines. A) Apototic ratio and mean sperm counts in X-miRBAC lines 1, 2 and 3, as assayed by analysis of two individuals of each genotype. Apoptotic ratios were calculated by dividing the number of TUNEL positive cells across multiple tubules of defined stage by the number of Sertoli cells across the same tubules. X-miRBAC line 1 exhibits apoptosis at stages IV and XII, X-miRBAC line 2 at stage XII and X-miRBAC line 3 at stage IV. B) Low power testis sections showing examples of TUNEL positive cells in defined tubule substages (rectangled). N.A means not applicable, as the line in question does not exhibit arrest at the particular tubule substage.
(EPS)

S1 Table List of amino-allyl oligonucleotides used. (TIF)

S2 Table Fold-change expression changes for individual pri-miRNAs in Spo11 null versus wild type, 15.5d*pp* testis. (TIF)

S3 Table Primer sequences. (TIF)
