# Supplementary figures and images for: Silencing of X-Linked MicroRNAs by Meiotic Sex Chromosome Inactivation
Source: PLoS Genet. 2015 Oct 28;11(10):e1005461. doi: 10.1371/journal.pgen.1005461 (PMC4624941; doi:10.1371/journal.pgen.1005461)

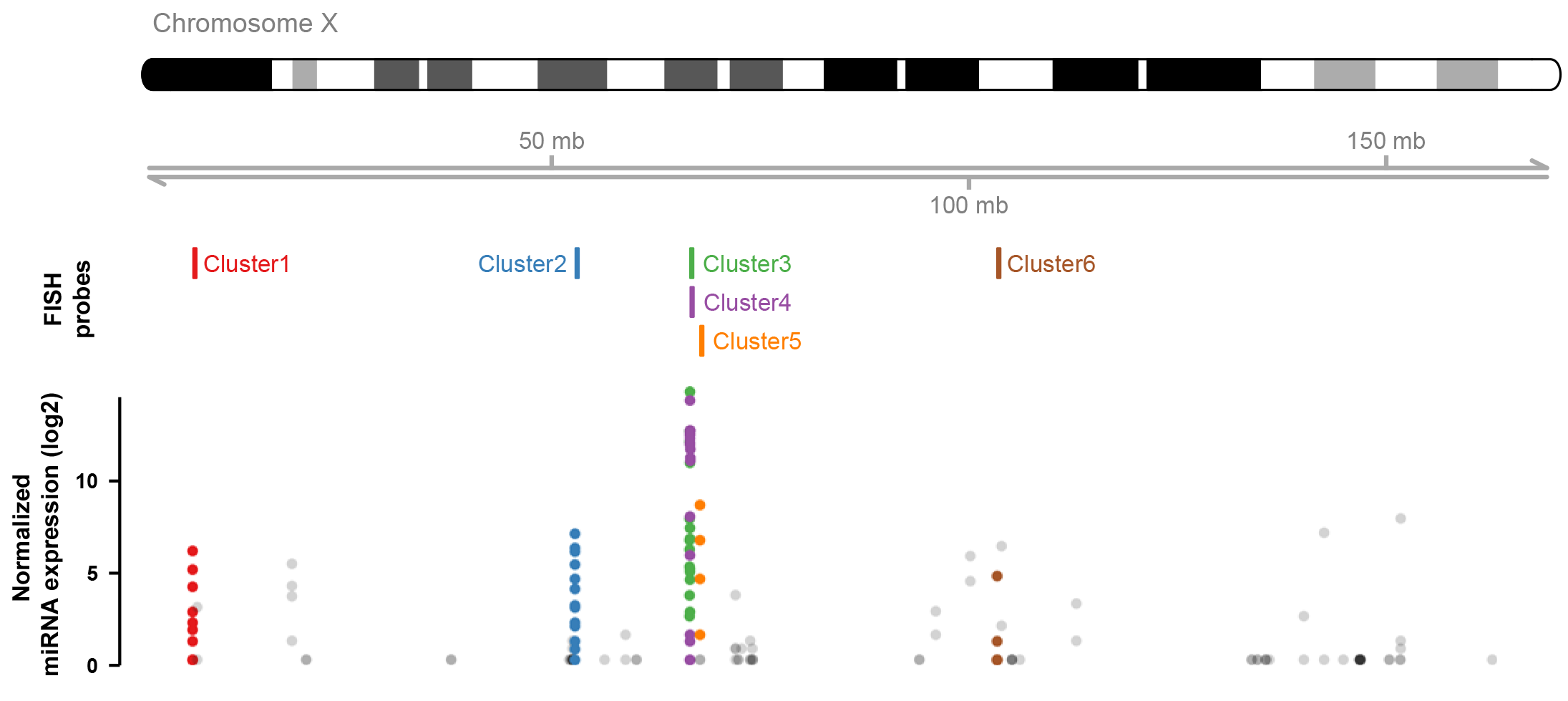

Supplement: S1 Fig — Normalized expression of X-linked miRNAs detected by RNA-sequencing in the testis are plotted against their genomic coordinates. Dots correspond to individual miRNAs. Positions of the fosmid probes used in RNA FISH are shown on top. The miRNAs targeted by RNA FISH are coloured by cluster. MiRNAs outside the clusters are in semi-transparent grey (overlapping miRNAs appear as darker data points). (TIF) [file pgen.1005461.s001.tif]

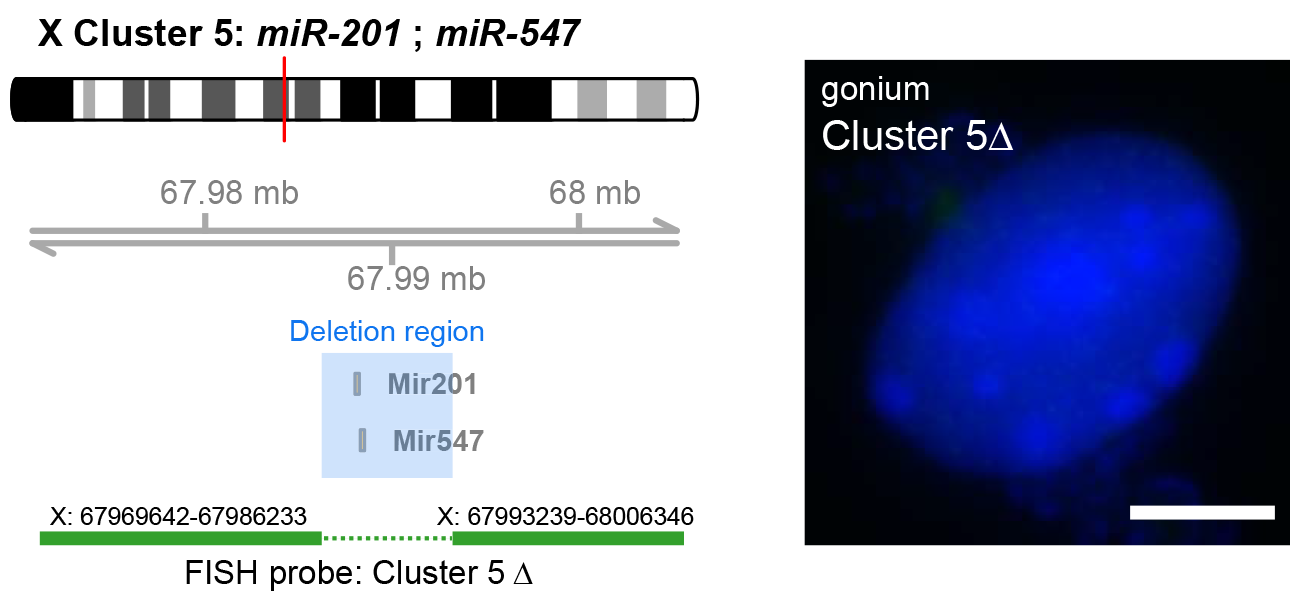

Supplement: S3 Fig — Top: Fosmid probe used for the detection of cluster 5 miRNA precursors by RNA FISH (probe 'Cluster 5'). A probe deleted for the miRNA genes was generated by recombineering (probe 'Cluster 5 Δ'). Bottom: a positive RNA FISH signal is detected with the original fosmid probe in spermatogonia (left panel, arrow). No signal is detected with the deletion probe (right panel). The RNA FISH experiment was performed in parallel with that of fig 1E. Green: RNA FISH signal, blue: DAPI. Scale bar: 5μm. (TIF) [file pgen.1005461.s003.tif]

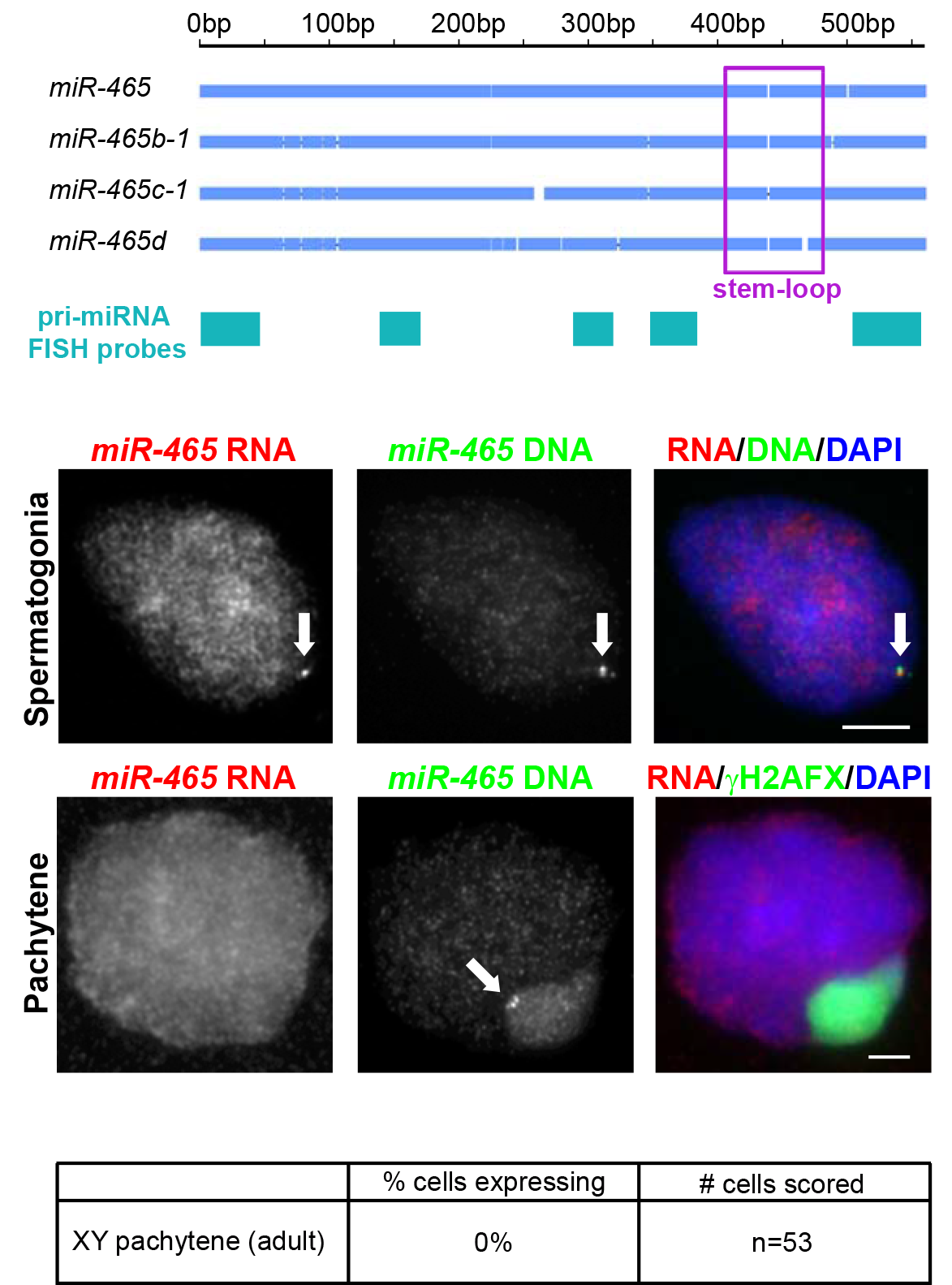

Supplement: S4 Fig — Top: Four variants of the miR-465 gene are repeated in the genome at the XA7 locus (see Fig 1D). Global DNA alignment of the precursors of the miR-465 variants is shown. Areas of high similarity (90% or more) are displayed in blue (source: CloneManager). Five pri-miRNA FISH probes were designed to target conserved sequences at the base of miRNA-containing stem-loop sequence. Middle: All five pri-miRNA probes were mixed for detection of pri-miRNA transcripts by FISH. BAC probes were used for DNA FISH. FISH signals are indicated by arrows. MiR-465 precursors were not detected in pachytene cells. MiR-465 expression pattern recapitulates that of cluster 4 miRNAs (Fig 1D). Note that pri-miRNA FISH signals were weaker than those obtained by fosmid RNA FISH. Scale bars: 5 μm. Bottom: Quantitative analysis of pri-miRNA FISH data for pachytene cells. (TIF) [file pgen.1005461.s004.tif]

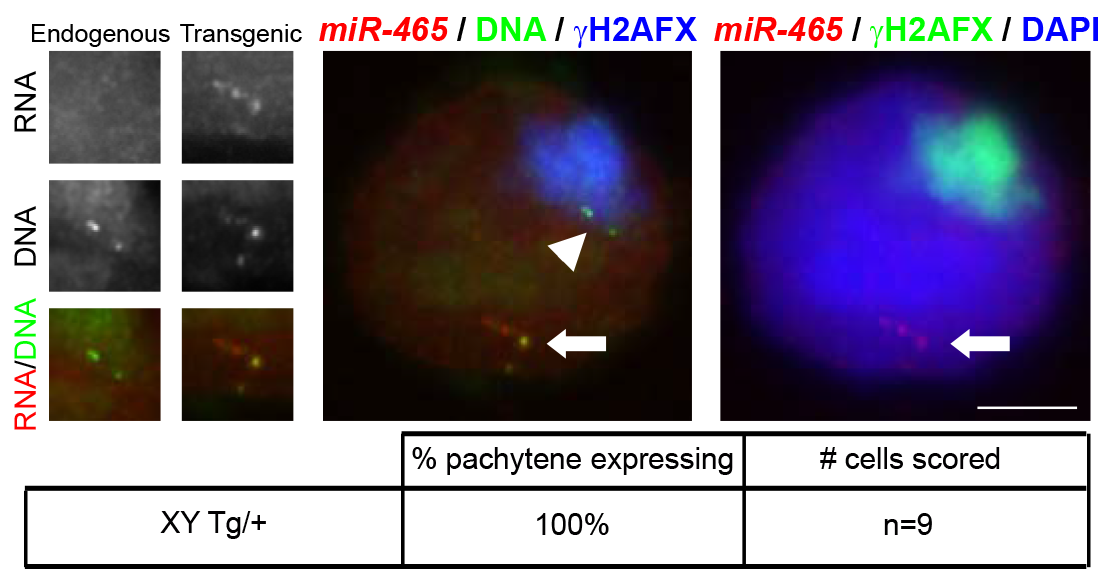

Supplement: S5 Fig — Combined DNA / pri-miRNA FISH for miR-465 shows expression of miR-465 from the autosomal, transgenic locus (arrows) but not from the X locus (arrowhead). Both the transgenic (arrows) and endogenous loci (arrowhead) are detected by DNA FISH. The insets show enhanced images of the endogenous and transgenic loci. Scale bar: 5 μm. (TIF) [file pgen.1005461.s005.tif]

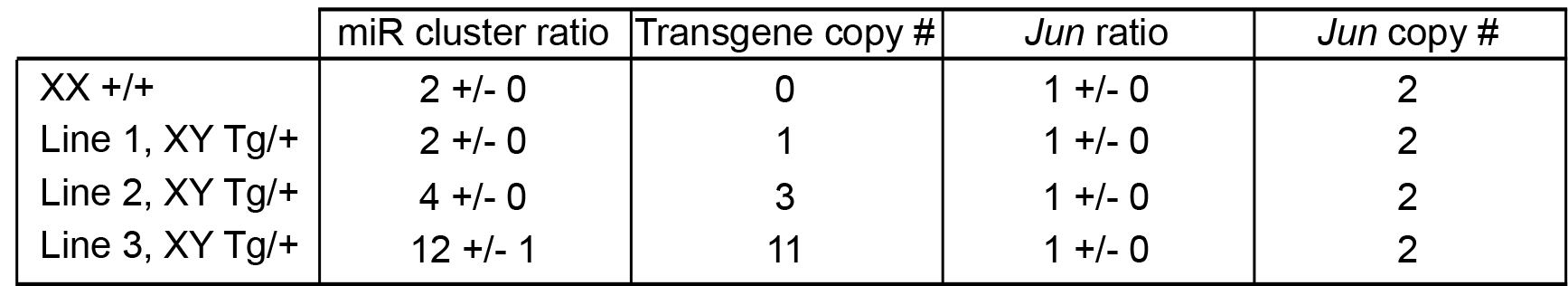

Supplement: S6 Fig — The number of copies of the miRNA region spanned by the transgene was estimated by qPCR on genomic DNA of heterozygous transgenic males. As a control, quantification was also made in a female (known number of copies of two). The transgene copy number was first expressed as the ratio between the number of copies in the tested sample (XX control or XY transgenic) and in an XY male. Results are expressed as the average +/- standard deviation of values obtained from three different genomic DNA dilutions. The number of copies of the transgene was then estimated by subtracting the number of copies of the miRNA cluster by the number of endogenous copies (one copy for XY, two for XX). As an additional control, we quantified the number of copies of the autosomal gene Jun (known copy number of two). (TIF) [file pgen.1005461.s006.tif]

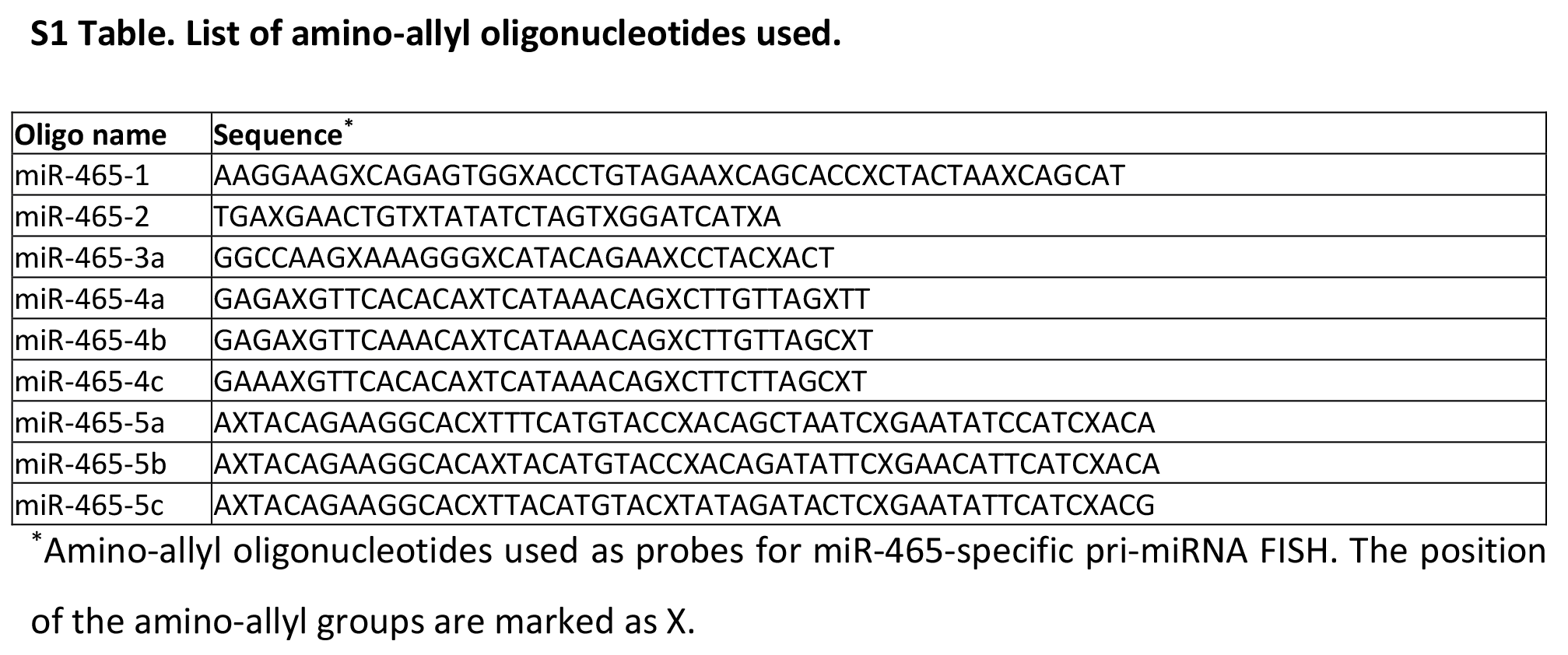

Supplement: S1 Table — (TIF) [file pgen.1005461.s008.tif]

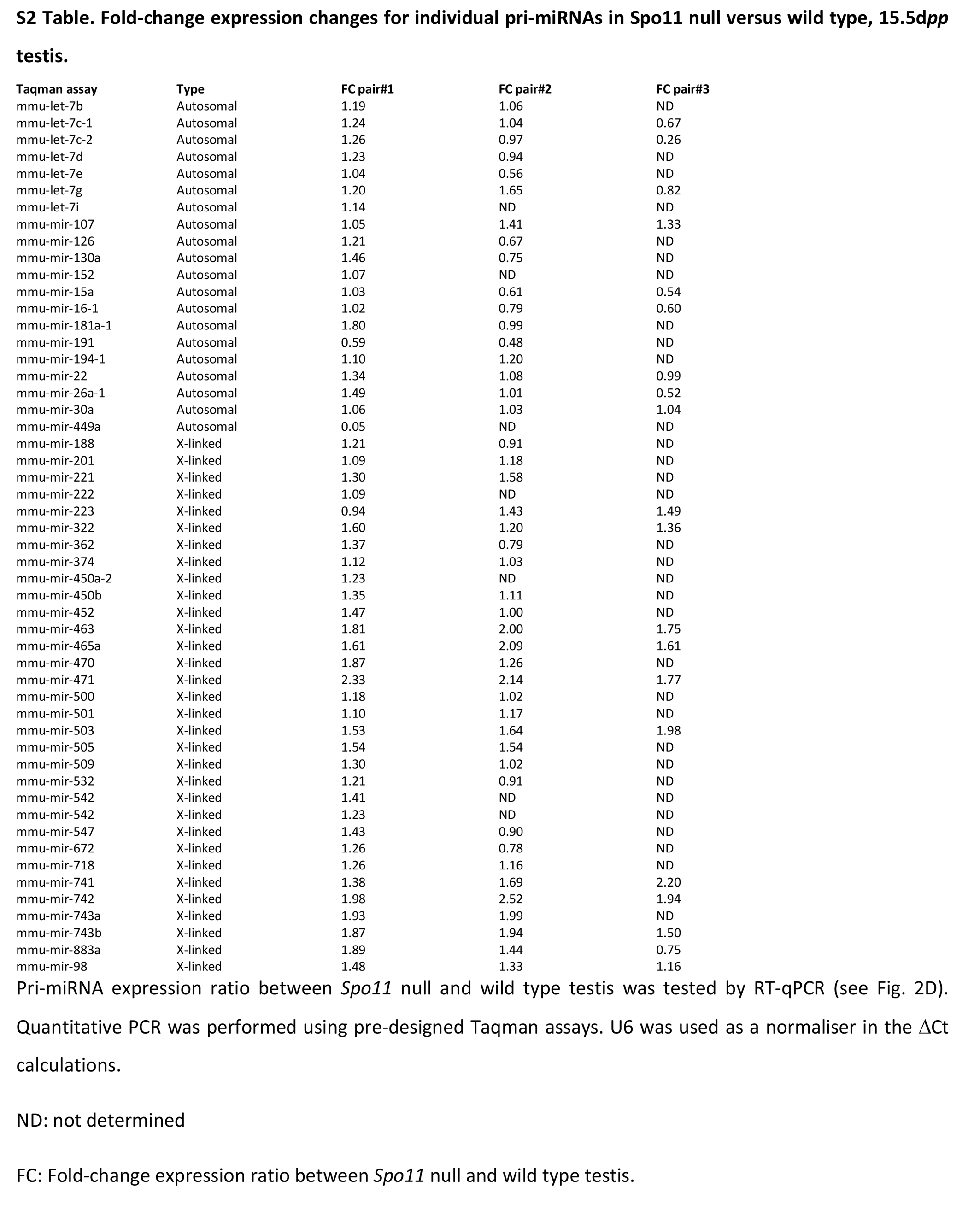

Supplement: S2 Table — (TIF) [file pgen.1005461.s009.tif]

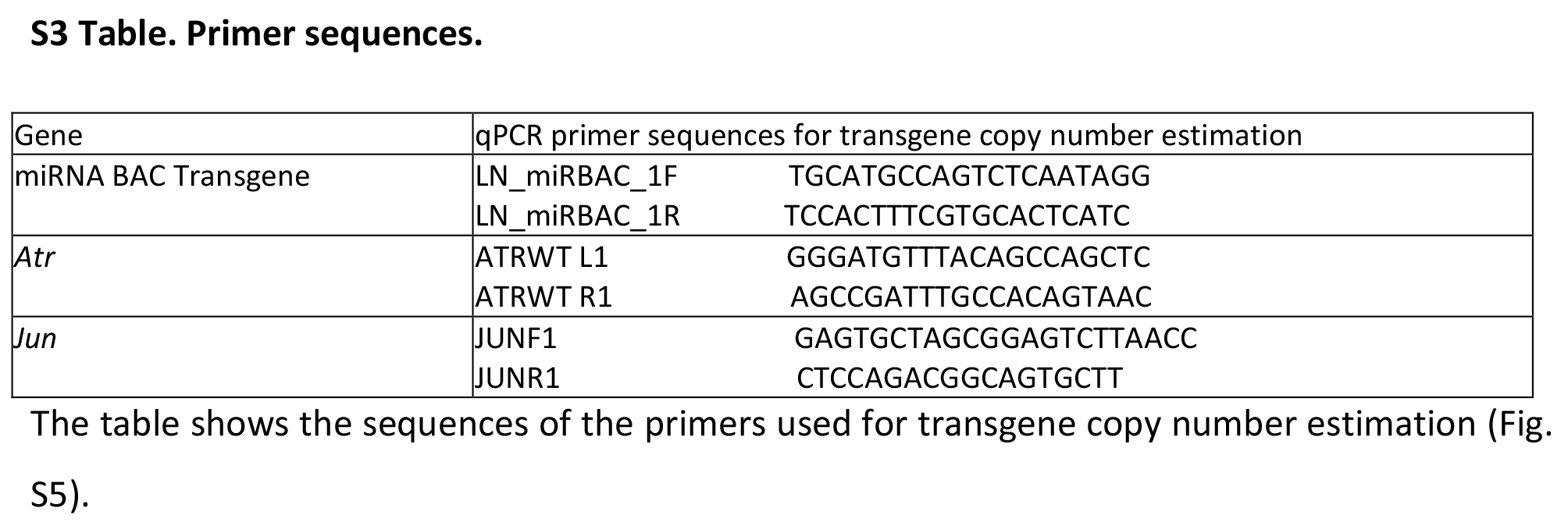

Supplement: S3 Table — (TIF) [file pgen.1005461.s010.tif]
